# Results of the IROCA international clinical audit in prostate cancer radiotherapy at six comprehensive cancer centres

**DOI:** 10.1038/s41598-021-91723-0

**Published:** 2021-06-10

**Authors:** Carla Lopes de Castro, Magdalena Fundowicz, Alvar Roselló, Josep Jové, Letizia Deantonio, Artur Aguiar, Carla Pisani, Salvador Villà, Anna Boladeras, Ewelina Konstanty, Marta Kruszyna-Mochalska, Piotr Milecki, Diego Jurado-Bruggeman, Joana Lencart, Ignasi Modolell, Carles Muñoz-Montplet, Luisa Aliste, Maria Gloria Torras, Montserrat Puigdemont, Luísa Carvalho, Marco Krengli, Ferran Guedea, Julian Malicki

**Affiliations:** 1 Instituto Português de Oncologia, Porto, Portugal; 2 Department of Electroradiology, Poznan University of Medical Sciences, Poznan, Poland; 3 Università degli Studi del Piemonte Orientale (UNIUPO), Novara, Italy; 4 Institut Català d’Oncologia, Badalona, Spain; 5 Institut Català d’Oncologia, Girona, Spain; 6 Institut Català d’Oncologia, L’Hospitalet, Barcelona, Spain; 7 Greater Poland Cancer Centre, Poznan, Poland; 8 Department of Radiation Oncology, Instituto Português de Oncologia Francisco Gentil-Porto, Porto, Portugal

**Keywords:** Cancer therapy, Urological cancer

## Abstract

To assess adherence to standard clinical practice for the diagnosis and treatment of patients undergoing prostate cancer (PCa) radiotherapy in four European countries using clinical audits as part of the international IROCA project. Multi-institutional, retrospective cohort study of 240 randomly-selected patients treated for PCa (n = 40/centre) in the year 2015 at six European hospitals. Clinical indicators applicable to general and PCa-specific radiotherapy processes were evaluated. All data were obtained directly from medical records. The audits were performed in the year 2017. Adherence to clinical protocols and practices was satisfactory, but with substantial inter-centre variability in numerous variables, as follows: staging MRI (range 27.5–87.5% of cases); presentation to multidisciplinary tumour board (2.5–100%); time elapsed between initial visit to the radiation oncology department and treatment initiation (42–102.5 days); number of treatment interruptions ≥ 1 day (7.5–97.5%). The most common deviation from standard clinical practice was inconsistent data registration, mainly failure to report data related to diagnosis, treatment, and/or adverse events. This clinical audit detected substantial inter-centre variability in adherence to standard clinical practice, most notably inconsistent record keeping. These findings confirm the value of performing clinical audits to detect deviations from standard clinical practices and procedures.

## Introduction

High dose ionizing radiation requires strict quality assurance to ensure patient safety and satisfactory clinical outcomes^1^. In recent years, there has been a growing interest^2,3^ in clinical audits to evaluate adherence to pre-defined quality indicators and clinical protocols^4,5^. However, to date, only a limited number of multi-institutional clinical audits have been conducted in the field of radiation oncology^2–4,6–8^.

Given the importance of quality assurance in radiotherapy, six comprehensive cancer centres in Europe initiated a multicentric systematic clinical audit project to improve the quality of radiotherapy. The primary aim of this project—known IROCA (*Improving Quality in Radiation Oncology through *Clinical Audits; https://iroca.eu)^9^ was to develop and apply a clinical audit model to assess adherence to core quality indicators to evaluate the key radiotherapy processes related to planning and delivery.

In the present article, we describe the results of the clinical audit for prostate cancer (PCa) radiotherapy at these six institutions. In this study, we assessed adherence to clinical protocols and international guidelines for diagnosis, treatment, and follow-up.

## Methods

This was a multi-institutional, retrospective cohort study involving patients (40 patients/centre) diagnosed with PCa at six European hospitals: the Wielkopolskie Centrum Onkologii (WCO) in Poznan, Poland; the Instituto Português de Oncologia (IPO) in Porto, Portugal; the Università degli Studi del Piemonte Orientale (UNIUPO) in Novara, Italy; and the three hospitals that form the Institut Català d'Oncologia (ICO) in Spain: ICO-Hospitalet [ICO-H], ICO-Badalona [ICO-B], and ICO-Girona [ICO-G]). The study was reviewed and approved by the ethics review boards at all participating institutions: the Clinical Research Ethics Committee (CEIC) of the Bellvitge University Hospital (which includes approval for three hospitals: ICO-H, ICO-B, and ICO-G); the Ethics Committee of the Portuguese Institute of Oncology, Porto; the Medical Ethics Committee of the Azienda Ospedaliero-Universitaria Maggiore della Carita; and the Ethics Committee of the Wielkopolskie Centrum Onkologii. As this was a retrospective study and all patient data were anonymised, written informed consent was waived at the time of ethics approval. The study was conducted in full accordance with the standard practices and the relevant national laws in the countries of the participating centres and with the principles of the Declaration of Helsinki.

### Design

Quality and clinical indicators were selected by a working group of radiation oncologists and medical physicists from the participating centres, who also developed the clinical audit model, which was broadly based on models used in previous clinical audits^2,10^. The clinical audit was performed to assess adherence to standard clinical practice for the study indicators. These consensus-based standards were defined by the team members after a review of the relevant guidelines (see below).

### Selection of quality indicators and clinical parameters

First, the working group reviewed the relevant literature^3,11,12^, including the main clinical guidelines (NCCN and EAU) for staging and treatment, as well as institutional and national guidelines. Based on this review and on our previous experience in performing clinical audits^2,10^, we selected a set of clinical indicators most relevant to PCa (supplementary Table S1). Three key phases of care were assessed: (1) diagnosis and pre-treatment: multidisciplinary tumour board (MTB); clinical record keeping; diagnostic tests; clinical trials; department clinical sessions; time between first visit and radiotherapy administration; (2) treatments administered: brachytherapy [BT]; external beam radiotherapy [EBRT]; dose/fractionation and treatment duration; treatment delays, interruptions, and compensations; radiotherapy and image-guidance techniques; adjuvant treatment; and (3) follow-up: presence and registration of adverse effects (AE), and follow-up adequacy. Toxicity was assessed according to the National Cancer Institute Common Terminology Criteria for Adverse Events (CTCAE), v. 4.0.

### Clinical audit

The clinical audit was performed from June to September 2017 by two external evaluators unaffiliated with the participating centres. All data were obtained from patient medical records and entered into a centralized online database.

### Target population and sample selection

The clinical records of 40 patients per centre (n = 240) who underwent primary radiotherapy for PCa in the year 2015 were reviewed. Inclusion criteria were: (1) confirmed diagnosis of PCa (ICD-9-CM code: 185; ICD-10-CM code: C61) and (2) curative-intent treatment (EBRT and/or BT). Exclusion criteria were: (1) absence of radical radiotherapy treatment, (2) presence of recurrent disease at diagnosis, and/or (3) presence of other cancers except non-melanoma skin cancer.

The sample size was estimated by assuming a reference proportion of 50% for any variable, with a minimum difference between two hospitals defined as 25%, with an alpha risk of 0.05 and beta of 0.10. The resulting sample size per hospital was 40 cases.

### Patient selection

All patients who met the inclusion criteria were assigned an identification number and then randomly selected for participation. After inclusion in the study, the selected patients were then stratified by risk groups (NCCN criteria), as follows: low-risk (≤ stage T2a and Gleason score [GS] ≤ 6 and PSA < 10 ng/mL); intermediate-risk (T2b-T2c or GS 7 or PSA 10–20 ng/mL), and high-risk (≥ T3a or GS ≥ 8 or PSA > 20 ng/mL or N+). Patients with multiple adverse intermediate risk factors were not included in the high-risk group if they did not present any high risk criteria.

### Statistical analysis

The Chi square test for categorical variables was used to compare results among hospitals. Fisher's exact test was used when frequencies were < 5. The SPSS-IBM statistical software program, v.21 (IBM, Armonk, NY; USA) and R software version 3.6.3 were used to perform the statistical analyses.

## Results

Table 1 shows the sample characteristics. Most patients (n = 138; 57.5%) were between 66 and 75 years of age. The biopsy report was included in the medical record in 97.9% of cases. Most patients (60.4%) underwent staging MRI of the prostate gland and pelvic lymph nodes, although inter-centre variability was wide (range 27.5–87.5%). CT scans were performed in 40.4% of the patients, mainly high risk ones, and some of these underwent PET scan (data not available). Bone scans were performed in 58.3% of the patients (inter-centre range 47.5–67.5%). Overall, 51.7% (n = 124) of patients were presented to the MTB, with significant (p < 0.001) inter-centre variation (range 2.5–100%). Two institutions failed to indicate on the medical records whether the patients were presented to the MTB.

**Table 1:** Characteristics of the sample (n = 240) by institution.

Hospital	ICO-B		ICO-G		ICO H		IPO		NO		WCO		All		P*
	n		n		n		n		n		n		n	%	
**Age at first fraction**															
< 65	10	25.00%	8	20%	9	22.50%	15	37.50%	4	10.00%	9	22.50%	55	22.92	< 0.001
66–75	27	67.50%	19	47.50%	28	70.00%	19	47.50%	19	47.50%	26	65.00%	138	57.50	
> 75	3	7.50%	13	32.50%	3	7.50%	6	15.00%	17	42.50%	5	12.50%	47	19.58	
All	40	100.00%	40	100.00%	40	100.00%	40	100.00%	40	100.00%	40	100.00%	240	100.00	
Diagnosed at a different hospital	23		33		29		39		32		40		196	81.7	0.9994
Biopsy report included in the medical record	39		39		40		40		37		40		235	97.9	0.137
**T stage**															
< T2a	13	32.50%	6	15.00%	21	65.63%	17	42.50%	22	55.00%	19	47.50%	98	42.24	
T2b-T2c	13	32.50%	8	20.00%	1	3.13%	11	27.50%	12	30.00%	1	2.50%	46	19.83	< 0.001
> T3a	11	27.50%	23	57.50%	10	31.25%	11	27.50%	6	15.00%	20	50.00%	81	34.91	
T2	3	7.50%	3	7.50%	–		1	2.50%	–		–		7	3.01	
All	40	100.00%	40	100.00%	32	100.00%	40	100.00%	40	100.00%	40	100.00%	232	100.00	
Missing					8								8	3.33	
< 10	24	60.00%	25	62.50%	26	65.00%	28	70.00%	30	75.00%	22	55.00%	155	64.58	
**PSA value**															
10–20	5	12.50%	7	17.50%	12	30.00%	4	10.00%	5	12.50%	10	25.00%	43	17.92	0.0189
> 20	11	27.50%	8	20.00%	2	5.00%	8	20.00%	5	12.50%	8	20.00%	42	17.50	
All	40	100.00%	40	100.00%	40	100.00%	40	100.00%	40	100.00%	40	100.00%	240	100.00	
< 6	9	22.50%	10	25.00%	19	47.50%	12	30.00%	13	32.50%	20	50.00%	83	34.58	
**Gleason score**															
3 + 4	10	25.00%	17	42.50%	7	17.50%	14	35.00%	11	27.50%	10	25.00%	69	28.75	0.0119
4 + 3	8	20.00%	3	7.50%	7	17.50%	4	10.00%	3	7.50%	8	20.00%	33	13.75	
> 8	13	32.50%	10	25.00%	7	17.50%	10	25.00%	13	32.50%	2	5.00%	55	22.92	
All	40	100.00%	40	100.00%	40	100.00%	40	100.00%	40	100.00%	40	100.00%	240	100.00	
Yes	21	52.50%	35	92.11%	24	61.54%	30	75.00%	24	60.00%	11	27.50%	145	61.18	
**Staging MRI**															
No	19	47.50%	3	7.89%	15	38.46%	10	25.00%	16	40.00%	29	72.50%	92	38.82	< 0.001
All	40	100.00%	38	100.00%	39	100.00%	40	100.00%	40	100.00%	40	100.00%	237	100.00	
Missing	0		2		1		0	0			0		3	7.50	
**Case presented to the MTB**															
Yes	6	35.29%	10	100.00%	29	78.38%	38	95.00%	1	2.50%	40	100.00%	124	67.39	< 0.001
No	11	64.71%	0	0.00%	8	21.62%	2	5.00%	39	97.50%	0	0.00%	60	32.61	
All	17	100.00%	10	100.00%	37	100.00%	40	100.00%	40	100.00%	40	100.00%	184	100.00	
Missing	23		30		3		0		0		0		56	23.33	

*MTB* multidisciplinary tumor board, *PSA* prostate specific antigen, *WCO* Wielkopolskie Centrum Onkologii, *IPO* Instituto Português de Oncologia (IPO) in Porto, Portugal, *IPO* Università degli Studi del Piemonte Orientale, *ICO-H* Institut Català d'Oncologia, Hospitalet, Spain, *ICO-B* (Badalona), *ICO-G* (ICO-Girona).

*Fisher’s exact test.

Risk group classification (NCCN criteria) was as follows: high risk (n = 120; 50%), intermediate risk (31.7%), and low risk (18.3%) (supplementary Figure S1).

Of the 240 patients, 4.2% (range 0–15%) were included in clinical trials (supplementary Table S2). Overall, most cases (62.5%) were presented at the departmental clinical session prior to treatment initiation; however, inter-institutional variability was large. Two centres (ICO-B and ICO-G) only presented selected cases at the departmental session.

Overall, median time between the first visit to the radiation oncology department and treatment initiation (EBRT or BT) was 78 days (range 42–102.5). Median time between CT simulation and radiotherapy was 18 days (range 12–33).

Most patients (87.1%, n = 209) received EBRT+/− a BT boost (n = 22). The remaining 31 patients (12.9%) received BT alone (supplementary Figure S2).

A wide range of doses were administered to the prostate in patients undergoing EBRT (supplementary Figure S3), mainly due to the use of different treatment approaches and combinations, which vary according to the risk category (low, intermediate, high).

The dose per fraction (Gy) in patients treated with EBRT is shown in supplementary Figure S4. Dose constraints were met in all cases unless otherwise indicated. Three of the six participating centres offered BT (supplementary Table S3). Fifty-three patients received BT, either HDR or LDR; in 17% of these patients, the dose rate was not specified in the medical record. Thirty-one patients received BT as monotherapy (145 Gy): 25 received 145 Gy LDR and six HDR-BT monotherapy (31.5 Gy/3 fractions). The other 22 patients received a single fraction (9, 10, or 15 Gy) HDR-BT boost.

**Figure 1:**
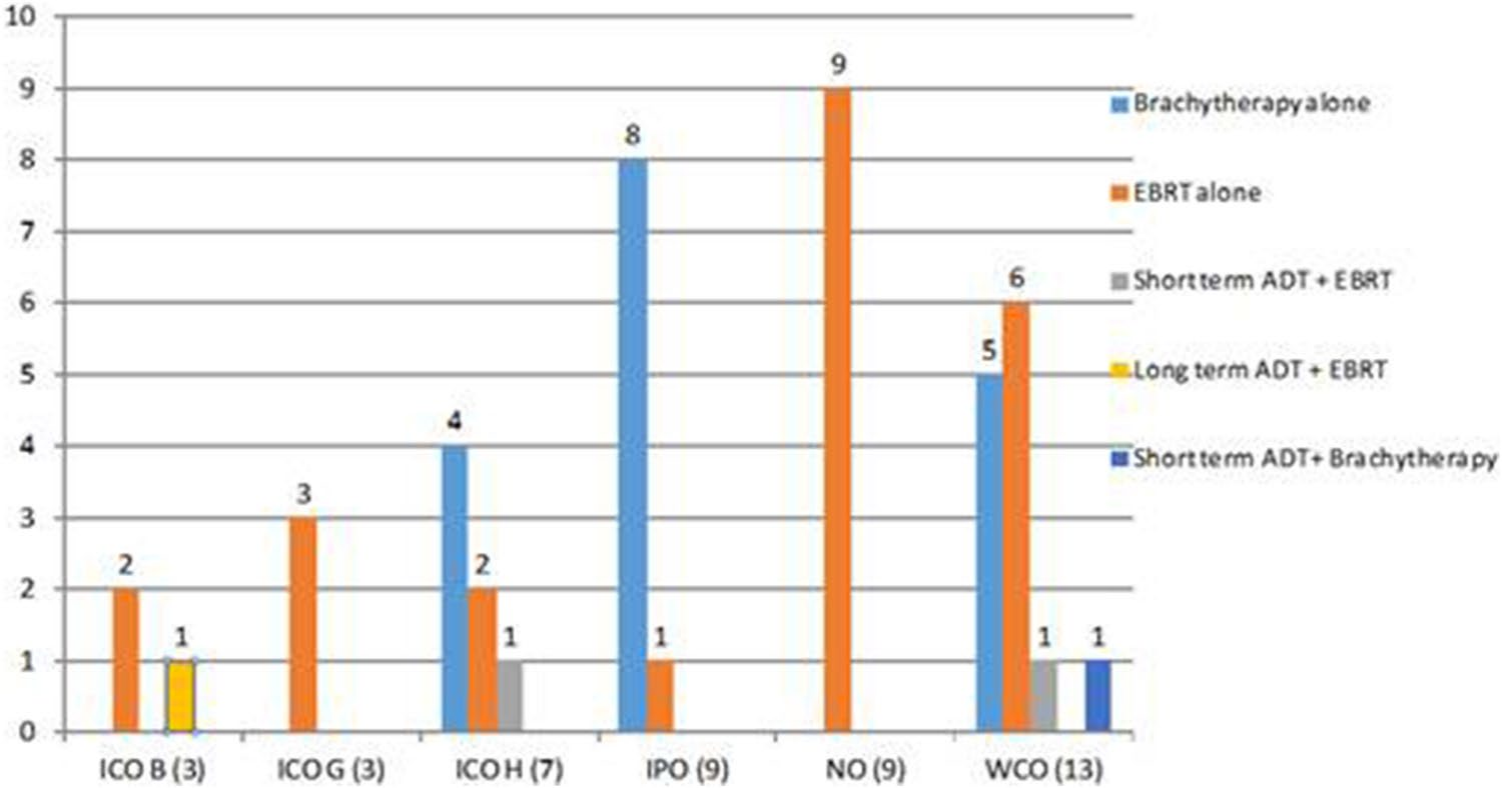
Treatment administered in low-risk patients and number of patients per treatment type.

**Figure 2:**
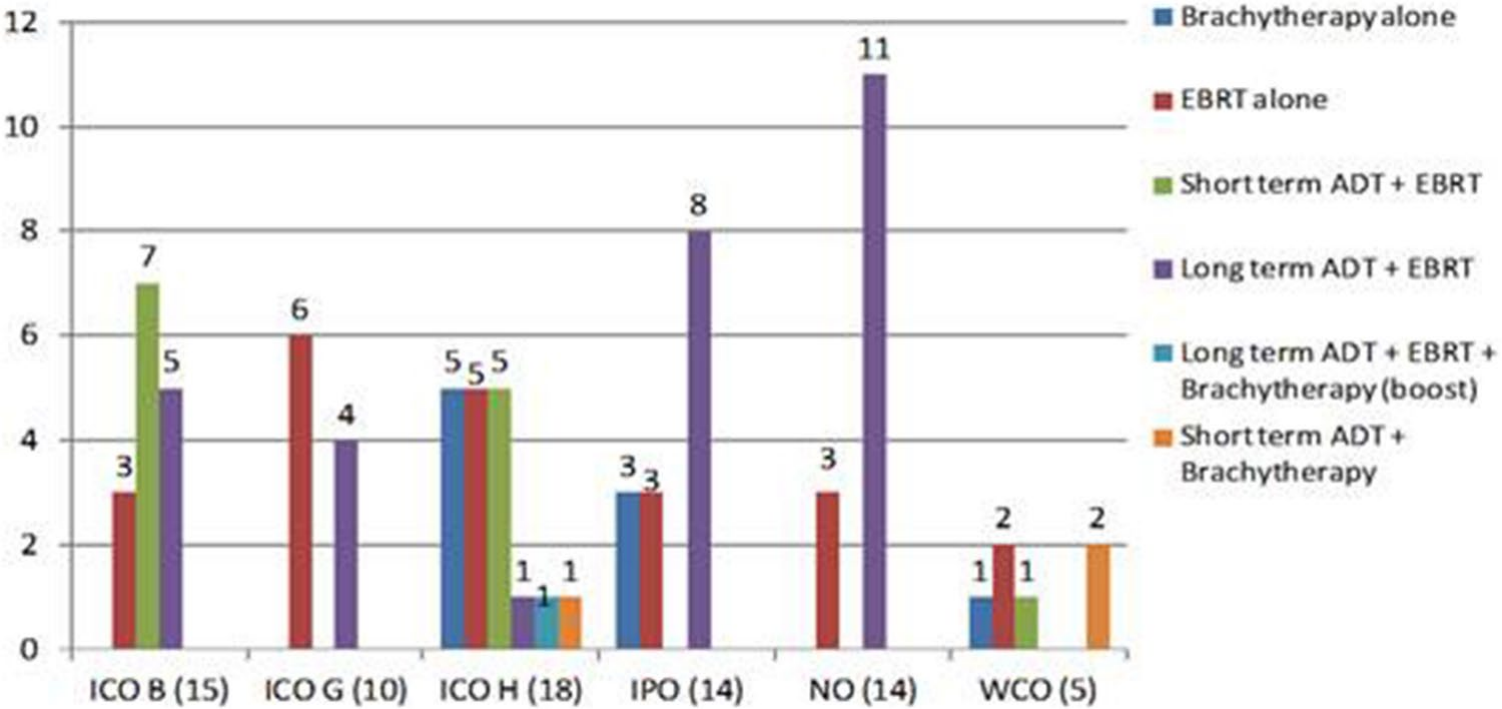
Treatment administered in intermediate-risk patients and number of patients per treatment type.

**Figure 3:**
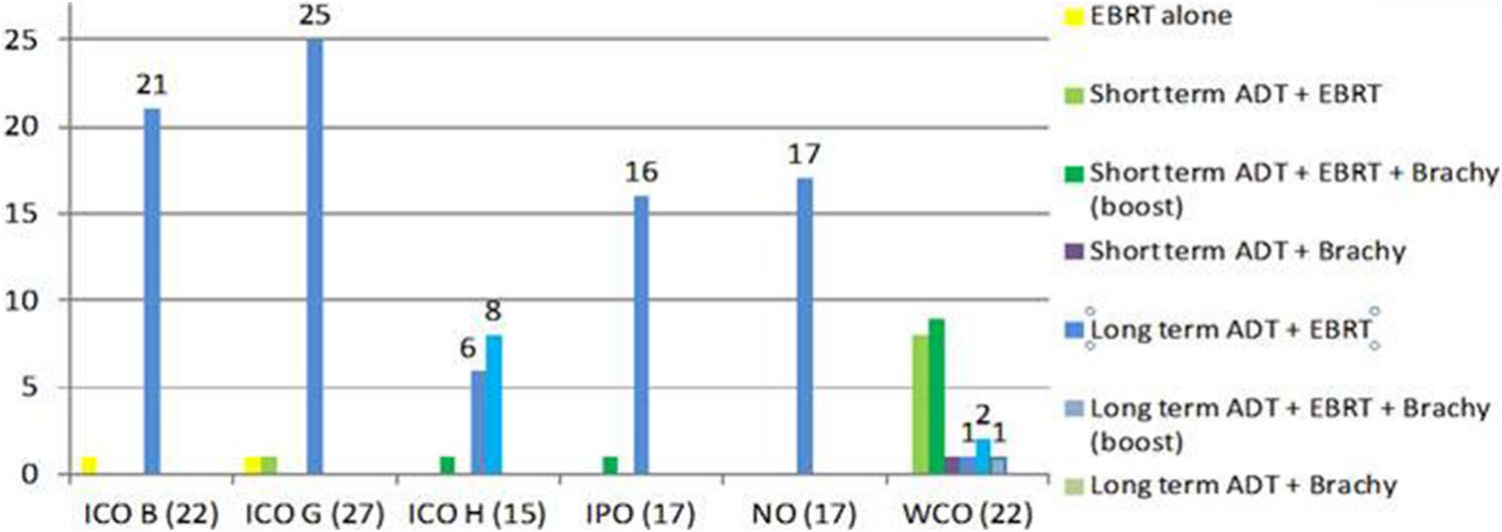
Treatment administered in high risk patients and number of patients per treatment type.

Figures 1, 2 and 3 show the treatment distribution according to risk group, revealing a wide range of different treatment regimens, consistent with routine variability in the radiotherapeutic treatment of PCa. As Fig. 1 shows, a range of different treatment regimens were used in the low-risk patients (n = 44). However, EBRT alone (n = 23; 52.3%) and BT alone (n = 17; 38.6%) accounted for 90.9% of all low-risk treatments. Only 4 patients received hormone therapy (HT).

Seventy-six patients (31.7%) had intermediate-risk PCa (Fig. 2). The most common treatment modalities in this risk group were long-term androgen-deprivation therapy (ADT) + EBRT (n = 29, 38.2%), followed by EBRT alone (n = 22, 28.9%), short-term ADT + EBRT (n = 13, 17.1%), and BT alone (n = 9, 11.8%). The remaining treatment options were used in only a limited number of cases. Intermediate-risk PCa, which comprises a heterogenous group of patients, is now divided into favourable and unfavourable intermediate-risk groups^13,14^. However, when these patients were treated, intermediate-risk patients were grouped into a single category (2016 NCCN criteria), and thus our intermediate group contains patients with both favourable and unfavourable risk characteristics, some of whom could be considered either unfavourable intermediate or high risk, depending on the clinical guidelines (i.e., NCCN or EUA).

Figure 3 shows the treatment distribution for high-risk patients. Exactly 50% of patients in this study (120/240) had high-risk PCa. The most common treatment regimens in the high-risk group were long-term ADT + EBRT (n = 86, 71.7%), long-term ADT + EBRT + BT boost (n = 10, 8.3%), and short-term ADT + EBRT + BT boost (n = 10, 8.3%). Only 2 patients (1.7%) received EBRT alone, probably due to the presence of comorbidities (e.g., cardiovascular disease) that contraindicated ADT (data not registered).

Treatment-related indicators are shown in supplementary Table S4. Treatment interruptions ≥ 1 day occurred in 67.1% of the patients treated with EBRT, ranging from 7.5 to 97.5% of cases, depending on the treating centre (p < 0.001).

Compensation rates for treatment interruptions were highly variable (range 0–87.5%), with some centres compensating for most or all interruptions while other centres either did not compensate for interruptions or data were not reported. Overall, 27.3% (range 5–72.4%) of patients completed treatment in the prescribed time; however, due to the relatively indolent nature of most cases of PCa, treatment interruptions of ≤ 4 days are considered reasonable and unlikely to affect the outcomes^15^. The percentage of patients completing treatment in the prescribed time ± 4 days was 67% (range 43.3–100%).

Most patients (79.9%) were treated with intensity-modulated radiotherapy (IMRT) or volumetric modulated arc therapy (VMAT). Only two centres performed SBRT. The remaining 20% of patients underwent three-dimensional radiotherapy (3DRT), with most of these patients being treated (57.5% and 37.5%, respectively) at ICO-B and ICO-G.

For image guidance, we assessed whether image-guided radiotherapy (IGRT) was performed and the technique (cone-beam CT [CBCT], fiducial markers, and KV/MV). All methods except KV/MV were considered acceptable (22% of patients).

Three of the six centres ICO-H, IPO and WCO) performed BT; in two of these centres (ICO-H and WCO), the BT boost was performed in 54% of high-risk patients. A BT boost was administered in 17.5% (n = 21) of high-risk patients overall.

### Adverse effects

AEs in this sample are summarized in supplementary Table S5. All participating centres registered AEs, which were observed in 65.4% of patients. The remaining patients (34.6%) either experienced no AEs or these were not registered.

### Follow-up

Most patients (96.7%) were alive at last follow-up. Regular follow-up (defined as ≥ 2 visits per year) was performed in nearly all patients (97.9%).

## Discussion

The present international, multi-institutional clinical audit was performed to compare adherence to standard clinical practice for prostate radiotherapy across six European cancer centres. Based on the predefined audit criteria, the participating centres generally adhered to standard clinical practice for diagnosis, treatment, and follow-up of PCa. Nonetheless, there was substantial inter-centre variability in clinical practice and several minor process-related deficiencies were detected. The key findings of the clinical audit are presented in Table 2 and discussed below.

**Table 2:** Recommended steps for improvement and/or harmonization selected indicators/processes.

Process or indicator	Finding	Recommended action
**Diagnostic and pre-treatment phase**		
Presentation to MTB	51.7% of cases presented to MTB Data missing in 23.3% of cases	Present all cases to MTB Implement quality control measures to ensure registration of data in 100% of cases
Presentation at departmental clinical session	62.5% of cases presented	Establish clear criteria for presenting cases to clinical sessions Consider using mini-tumor boards comprised of radiation oncologists specializing in the specific tumor type/location
Staging MRI	60.4% of cases (range 27.5–87.5%)	Implement measures to increase to 100%
Participation in clinical trial	4.2% (range 0–15%)	At centers with low rates, seek to raise percentage of patients participating in clinical trials
**Treatment phase**		
Median time elapsed between first visit and start of radiotherapy	78 days (range 42–102.5)	Implement measures to reduce this time interval
Median time interval between CT simulation and RT	18 days (range 12–33)	Apply measures to reduce this time interval
Treatment interruptions	67% of cases (EBRT), range 7.5–97.5%	Implement measures to reduce interruptions at centers with a high percentage of treatment interruptions Consider working on Saturdays and holidays
Treatment compensation rates	9.9% of cases compensated (range 0–87.5%) Data not registered in some cases	Implement measures to increase compensation rates Apply quality control measures to ensure registration of data in 100% of cases
Treatment completed in prescribed time	67% of treatments completed in prescribed time ± 4 days (range 43.3–100%)	Apply procedures to raise rate to 100% Consider working on Saturdays and holidays
Long-term ADT in high-risk patients	80% of high risk cases received long term ADT	Ensure that 100% of high risk patients receive long term ADT
**Follow-up phase**		
Registration of AEs absent	Data missing in 34.6% of cases	Implement quality control measures to ensure registration of data in 100% of cases

*MTB* multidisciplinary tumor board, *MRI* magnetic resonance imaging, *CT* computed tomography, *ADT* androgen deprivation therapy, *AE* adverse events, *RT* radiotherapy.

Slightly more than half of the cases were presented to the MTB, with substantial inter-centre variation (2.5–100%). This finding was not unexpected given the lack of a generally-accepted protocol for presenting patients to a MTB, and these heterogenous findings appear to reflect variability in clinical practice across Europe. Due to missing data, we were unable to determine whether 56 cases (23.3%) had been presented to the MTB. Although this high number of missing cases suggests a failure to register key data, a large proportion of these “missing” cases are likely low-risk patients who underwent BT monotherapy, which are not routinely presented to the MTB in some centres. The value of presenting all cases to a MTB prior to treatment is supported by published reports showing that the diagnosis and/or treatment plan can change in up to 50% of cases after presentation to the MTB^16^. In a survey of patients with non-metastatic prostate cancer conducted by Patrikidou et al.^17^, multidisciplinary consultation improved patient satisfaction and strongly influenced the final treatment decision. However, the structure of the MTB and the expertise of the participants play a vital role in the value of a given MTB^18^. Considering the time and expense involved in routinely presenting patients to a MTB, some authors recommend “mini” tumour boards comprised of only a few selected experts when there is a lack of available expertise and/or specialists^19,20^.

Four of the six hospitals (66.7%) in this study presented all or nearly all patients at the department clinical session prior to treatment initiation. However, two of the centres reported presenting only complex or unique cases. During the post-audit meeting to discuss our findings, representatives from the participating institutions debated the value of routinely presenting all patients at the departmental session. The centres that do not regularly do so argued that heavy caseloads and time constraints make this impractical and—moreover—may not provide much value due to the scant time allotted to each individual case; in addition, if a large number of routine cases are presented, clinicians are less likely to pay attention. By contrast, other researchers argued that all cases should be presented as a quality control measure to detect errors and to help train residents. One centre takes an intermediate approach, presenting special cases to a “mini-board” comprised of specialists in that specific tumour site, an approach that offers several potential advantages: it avoids “information overload” at the departmental clinical sessions and ensures that physicians who evaluate the case have specific expertise in that tumour site. One key measure of quality is the inclusion of all relevant clinical data in medical record^21^. In patients with cancer, the biopsy report provides essential information. Unsurprisingly, we found that the biopsy report was included in the medical records of 98% of cases, indicating good adherence to quality among the participating institutions. Nevertheless, the few missing cases point to a minor but important deviation from good practice.

Most clinical guidelines^22^ recommend staging MRI for patients with PCa, although its value in low-risk patients (except in active surveillance) has been questioned^22–24^. Most patients (60.4%) in this study underwent staging MRI, but with wide inter-centre variability (27.5–87.5%), possibly due to difference in clinical practices. For example, the centre with the lowest proportion of patients who underwent staging MRI used an alternative technique (ultrasound) to stage patients with low-risk PCa and MRI/CT fusion in intermediate and high-risk patients. CT can be considered acceptable to detect metastatic disease, but not for prostate gland evaluation. Finally, although staging MRI has now become standard, during the study period (2015) many centres were still transitioning from CT.

Waiting times are among the most important quality variables, as excessive delays between diagnosis and treatment initiation can negatively impact treatment outcomes by increasing the risk of tumour growth and metastasis^25^. Although longer waits are unlikely to impact clinical outcomes in patients with low-risk PCa, this is not true in intermediate or high-risk cases, with the available evidence showing that excessive waiting times can negatively affect oncological outcomes^26^. For this reason, it is crucial to minimize waiting times whenever possible. We observed wide variability in the time elapsed from the first visit to the radiation oncology department to the start of radiotherapy (range 42–102.5 days). Several factors may account for these inter-institutional differences, including differences in patient management practices: in some centres, staging is performed by the radiation oncologist (which could delay treatment initiation) whereas in other centres the patient is referred to the radiation oncologist after staging has been performed. In some cases, treatment may be intentionally delayed when there is a clinical advantage in doing so (e.g., in patients with highly symptomatic disease, or in those who recently started HT). The median time interval between CT simulation and radiotherapy was 18 days (range 12–33 days). One centre presented a wider range (16–120 days), suggesting poor compliance with standard clinical practice, which recommends that the CT be repeated after ≥ 30 days if the patient has not—for any reason—initiated radiotherapy^22^.

Studies conducted to assess the causes of long waiting times suggest that lack of timely treatment may be related to inefficient healthcare processes, limited medical resources, and absence of policy support^27^. Some authors have suggested that these should be considered an indicator of access to healthcare^28^. Importantly, longer waiting times are often—although not always—associated with worse outcomes^29,30^. Wyatt et al. found that a delay of 1–2 months does not significantly impact tumour control rates in patients with PCa^31^. By contrast, Nakayama et al. found that delays > 6 months increased the risk of biochemical progression in patients with localized PCa^32^. Mackillop et al.^29^ concluded that delayed initiation of radiotherapy may be associated with a clinically-significant deterioration in local control, leading those authors to emphasize the importance of minimizing these times. In this context, our findings reveal that most, but not all, of the participating centres complied with clinical protocols for this indicator. Clearly, the centres that failed to comply with these recommendations will need to take measures to ensure that waiting times are within acceptable limits.

Our results show wide variability in treatments, even among patients in the same risk category. Most of the low-risk patients underwent single-modality therapy—either EBRT alone (52.3%) or BT alone (38.6%)—data that are in line with current clinical guidelines^33^. As might be expected, the widest variability in treatment selection was observed in the intermediate-risk group (Fig. 2), even though most (67%) of those patients were treated either with EBRT alone (28.9%) or EBRT + long-term ADT (38.2%). Nevertheless, this variability among intermediate-risk patients should be expected due to differences within this risk group, as some may be closer to low-risk (e.g., those who received EBRT alone) while others present features closer to high-risk patients. Intermediate-risk PCa is highly heterogeneous, displaying a wide range of clinical behaviour and aggressiveness, and biochemical and clinical recurrence rates can range from as low as 2% to as high as 70%^34,35^, which is why intermediate-risk patients are now generally subclassified into favourable vs. unfavourable risk groups to more accurately reflect the real risk of recurrence^33,36–38^.

Treatment interruptions are an important indicator of quality that can have a marked effect on outcomes^39^. The usual causes of interruptions include machine malfunction or maintenance, toxicity, holidays, or unplanned quality control checks, which is why it is essential to have in place protocols to determine how to compensate for the interruption. In most cases, definitive EBRT with conventional fractionation schemes for localized PCa requires 8 weeks of treatment, which means that treatment interruptions are common in these patients, even though brief interruptions do not appear to have a major impact on outcomes. Dong et al.^15^ found that even long treatment breaks (≥ 4 fraction) did not significantly affect survival outcomes in patients receiving high dose (> 74 Gy) radiation without ADT, provided that all missing fractions were delivered by the end of treatment. As those authors observed, those findings reflect the relatively indolent course of PCa compared to other cancers. Nonetheless, quality clinical practice dictates that all efforts should be made to avoid interrupting radical radiotherapy treatments; when interruptions are unavoidable, compensation is essential. According to the Royal College of Radiologists^40^, any interruption to radiotherapy schedules may affect the outcome and thus interruptions should be “as short as reasonably achievable”. Although prolongation up to 5 days may not be detrimental, no safe minimum has been established, which is why those guidelines recommend limiting treatment prolongation to no more than 2 days over the original prescription^2,40^.

This clinical audit revealed important differences among centres in treatment interruptions, raising the possibility that some institutions may need to re-assess quality control for this particular aspect. Treatment interruptions of 1 day or more were common, occurring in two-thirds of patients treated with EBRT, with significant variability among centres (7.5–97.5%). Similar inter-centre heterogeneity was observed with regard to treatment compensation rates. While some centres compensated for all (or nearly all) interruptions, others either did not do so or—importantly—data were not available. Compensation rates ranged from 0 to 87.5% (overall, 9.9%). Surprisingly, only slightly more than one-quarter of patients who underwent EBRT completed treatment in the prescribed time, with high variability among centres (5–72.4%). However, if we add 4 days to the expected overall treatment time (OTT)—a common practice in routine care—the percentage of patients completing treatment in the prescribed time rises to 67% (range 43.3–100%). Ideally, 100% of patients should complete treatment in the prescribed time and the 67% rate in this study points to a clear target for improvement. The centres with the highest percentage of treatment interruptions were in Spain and Italy, with the lowest in Poland and Portugal. While the reasons for these differences are not entirely clear, a recent single-institution study in Spain^41^ found that public holidays accounted for 45% of unscheduled interruptions, and these interruptions were associated with a lower probability of local disease control and/or an increase in the likelihood of biochemical failure in patients with PCa. Based on those findings, the authors concluded that outcomes of fractionated radiotherapy could be improved by working on public holidays. That same group also reported their experience in managing interruptions^42^, recommending two concrete measures to reduce OTT: working on public holidays and performing maintenance tasks on Saturdays. Those authors calculated that implementation of those two measures reduced the likelihood of biochemical failure by a mean of 2% (and by ≥ 4% in nearly 20% of patients). Other authors have also provided advice on how to manage unscheduled treatment interruptions in patients with PCa^43^.

Clinical recommendations call for long-term ADT (≥ 2 years) in high-risk patients. In our cohort, 96 of these 120 high-risk patients (80%) received long-term ADT, although inter-institutional variability was high, suggesting less than optimal adherence to clinical guidelines; however, because ADT is contraindicated in patients with certain conditions (e.g., severe cardiovascular disease), it seems likely that at least some of the patients who did not receive long-term ADT may have presented some type of contraindication.

We found that all centres registered AEs on the medical record, with 65.4% of patients experiencing AEs and the remaining cases (34.6%) either not presenting AEs or these events were not registered. Clinical guidelines stipulate that the presence or absence of side effects should be reported^22,23,33^. In this regard, it appears that AEs may have been underreported at some centres and/or differently graded. For example, one institution failed to indicate in 23 cases whether or not cystitis-urethritis was present. In addition, in 102 cases (42.5%), the participating institutions failed to report the present or absence of erectile dysfunction. This finding shows that only some of the acute AEs were registered on the medical record. The failure to register all relevant AEs (or their absence) highlights an area that should be targeted for improvement.

Nearly all patients (n = 235, 98%) received regular follow up (≥ 2 visits per year) but due to short follow-up and wide variability in risk groups, it was not possible to compare the outcomes among the participating centres.

Although we have sought to put the findings of the present audit in the context of other studies, it is difficult to compare results due to methodological differences, particularly due heterogeneity in the parameters evaluated. Comparison is further hindered by the scant data available and because the parameters must, of necessity, vary according to tumour type, as evidenced in the previous IROCA studies for prostate and rectal cancer^9,44^. In this regard, we still lack a set of internationally recognized, standardized indicators to permit international comparisons among radiotherapy centres^3^, despite efforts made by several different groups^3–5,11,45^.

Most clinical audits conducted to date in radiotherapy have been single-centre audits, many of which have been performed using the IAEA audit methodology known as Quality Assurance Team for Radiation Oncology (QUATRO). Izewska et al.^46^ explored the factors that impacted quality of care among QUATRO-audited centres (n = 34) in Europe. The main findings relevant to barriers to quality care were insufficient staffing, education/training, equipment, and lack of quality management. Few multi-institutional audits have been carried out to date, with the notable exception of those performed by our group and by Dutch and Belgian groups^6,7,47^. Aside from the IROCA project, one of the largest multi-centre audits was a national systematic clinical audit of all radiotherapy departments in Belgium (n = 25) from 2011 to 2015 using the IAEA QUATRO methodology. In that study, the authors analysed the clinical audit reports for the individual departments to identify the most common areas for improvement, which included the need for process improvement and protocol development (a recommendation found in 34% of the reports), deficiencies in human resources and physical infrastructure (27%), issues related to departmental organisation (20%), and no systematised approach to follow-up after radiotherapy delivery. Although those findings echo many of the findings in our audit, the methodological differences make these studies difficult to compare directly.

The main limitation of the present study is the sample size (40 patients/centre), thus limiting the statistical power of the analysis. Although this sample size is insufficient to assess clinical outcomes (e.g., survival), this was not a study aim. Rather, since our aim was to identify differences among the centres in adherence to key processes such as registration of clinical data, the sample size was sufficient to detect numerous aspects amenable to improvement and harmonization (Table 2). Another limitation was the omission of several potentially relevant variables and/or indicators such as some staging tests (e.g., pelvic CT and bone scintigraphy) and pelvic node irradiation, which were intentionally omitted from the analysis for feasibility reasons. Another limitation was the number of intermediate-risk patients (NCCN risk criteria) who were prescribed long-term ADT by the referring hospital, which impeded our assessment of adherence to standard clinical practice. Although we assessed whether IGRT was performed and the type of IGRT, we did not evaluate the frequency of verification, nor did we comprehensively investigate the method used (e.g., online vs. offline). The main strength of this study was the multi-institutional, multinational design with objective external auditors. Another strength is that, in an effort to minimize bias, we considered only objective data obtained from patient medical records.

The purpose of performing regular clinical audits is to continuously improve processes to obtain better outcomes. Research has shown that the mere act of identifying differences can induce participants to seek to harmonise processes to eliminate large deviations from the norms. Indeed, this was confirmed during the post-study discussions among the researchers, as the findings of the study clearly revealed numerous targets for improvement and/or harmonization.

## Conclusions

The present clinical audit, performed at six radiation oncology departments in four European countries, reveals generally good adherence to standard clinical practice. The wide variability of treatment approaches observed in this study reflects, in part, the substantial range of treatment options with comparable clinical outcomes in prostate cancer. However, some minor deviations from quality clinical practice were detected, most notably inconsistent data registration. We also observed inter-institutional differences in the presentation of cases to departmental clinical sessions and to the multidisciplinary tumour board, as well as differences in the approach to managing treatment interruptions.

A major benefit of this clinical audit is that it allowed us to identify and jointly discuss difference in clinical practice—some of which are justified—and areas of weakness, which should ultimately encourage the participating institutions to implement better practices, thus leading to greater harmonization among centres in the management of prostate cancer.

The IROCA clinical audit project may provide a useful template for other institutions to follow, potentially promoting a wider use of external clinical audits in radiotherapy. Despite the time and resources needed to conduct a thorough clinical audit, identifying and correcting deviations from standard clinical practice is essential to improving patient outcomes.

## Supplementary Information

Supplementary Information 1.

## Data Availability

The datasets used and/or analysed during the current study are available from the corresponding author on reasonable request.

## References

[1] Malicki J (2014). Patient safety in external beam radiotherapy—guidelines on risk assessment and analysis of adverse error-events and near misses: Introducing the ACCIRAD project. Radiother. Oncol..

[2] Fundowicz M (2014). Preoperative radiotherapy for rectal cancer: A comparative study of quality control adherence at two cancer hospitals in Spain and Poland. Radiol. Oncol..

[3] Cionini L (2007). Quality indicators in radiotherapy. Radiother. Oncol..

[4] Qian X (2011). IAEA human health series No. 4, comprehensive clinical audits of diagnostic radiology practices: A tool for quality improvement. Health Phys..

[5] Shortt K, Davidsson L, Hendry J, Dondi M, Andreo P (2008). International perspectives on quality assurance and new techniques in radiation medicine: Outcomes of an IAEA conference. Int. J. Radiat. Oncol..

[6] Ishikura S (2008). Quality assurance of radiotherapy in cancer treatment: Toward improvement of patient safety and quality of care. Jpn. J. Clin. Oncol..

[7] Kaur J, Mohanti BK, Muzumder S (2013). Clinical audit in radiation oncology: Results from one academic centre in Delhi, India. Asian Pac J. Cancer Prev..

[8] Scalliet PGM (2015). Clinical radiotherapy audits in Belgium, 2011–2014. Cancer Radiothér..

[9] Torras MG (2017). Improving radiation oncology through clinical audits: Introducing the IROCA project. Rep. Pract. Oncol. Radiother..

[10] Torras MG (2018). Clinical audit of the radiotherapy process in rectal cancer: Clinical practice guidelines and quality certification do not avert variability in clinical practice. Transl. Oncol..

[11] Gagliardi AR, Fleshner N, Langer B, Stern H, Brown AD (2005). Development of prostate cancer quality indicators: A modified Delphi approach. Can. J. Urol..

[12] Freeman AR, Roos DE, Kim L (2014). Quality indicators for prostate radiotherapy: Are patients disadvantaged by receiving treatment in a ‘generalist’ centre?. J. Med. Imaging Radiat. Oncol..

[13] Bracci S (2016). Different outcomes among favourable and unfavourable intermediate-risk prostate cancer patients treated with hypofractionated radiotherapy and androgen deprivation therapy. Radiat. Oncol..

[14] Rodrigues G (2012). Pre-treatment risk stratification of prostate cancer patients: A critical review. Can. Urol. Assoc. J..

[15] Dong Y (2018). Effects of interruptions of external beam radiation therapy on outcomes in patients with prostate cancer. J. Med. Imaging Radiat. Oncol..

[16] Charara RN (2017). Practice and impact of multidisciplinary tumor boards on patient management: A prospective study. J. Glob. Oncol..

[17] Patrikidou A (2018). Helping patients make informed decisions. Two-year evaluation of the Gustave Roussy prostate cancer multidisciplinary clinic. Clin. Transl. Radiat. Oncol..

[18] Keating NL (2013). Tumor boards and the quality of cancer care. J. Natl. Cancer Inst..

[19] El Saghir NS (2011). Survey of utilization of multidisciplinary management tumor boards in Arab countries. Breast.

[20] Anderson BO (2011). Optimisation of breast cancer management in low-resource and middle-resource countries: Executive summary of the Breast Health Global Initiative consensus, 2010. Lancet. Oncol..

[21] Newman JR (2015). Survival trends in hypopharyngeal cancer: A population-based review. Laryngoscope.

[22] Carroll PH, Mohler JL (2018). NCCN guidelines updates: Prostate cancer and prostate cancer early detection. J. Natl. Compr. Canc. Netw..

[23] Dell’Oglio P (2018). MP53–20 multi-institutional external validation of the Eau guidelines recommendations for the use of staging MPMRI prior to radical prostatectomy in men with intermediate and high-risk prostate cancer. J. Urol..

[24] Walton-Diaz A, Rais-Bahrami S (2018). When to order an MRI in the initial evaluation and management of prostate cancer. Oncology (Williston Park)..

[25] Chen Z, King W, Pearcey R, Kerba M, Mackillop WJ (2008). The relationship between waiting time for radiotherapy and clinical outcomes: A systematic review of the literature. Radiother. Oncol..

[26] Robertson S (2017). Waiting times for cancer patients in Sweden: A nationwide population-based study. Scand. J. Public Health.

[27] Atun R (2015). Expanding global access to radiotherapy. Lancet Oncol..

[28] Smith BD (2010). The future of radiation oncology in the United States from 2010 to 2020: Will supply keep pace with demand?. J. Clin. Oncol..

[29] Mackillop WJ, Ch MBB, Bates JHT, O’Sullivan B, Withers HR (1996). The effect of delay in treatment on local control by radiotherapy. Int. J. Radiat. Oncol. Biol. Phys.

[30] Hess CB, Chen AM (2014). Measuring psychosocial functioning in the radiation oncology clinic: A systematic review. Psychooncology..

[31] Wyatt RM, Beddoe AH, Dale RG (2003). The effects of delays in radiotherapy treatment on tumour control. Phys. Med. Biol..

[32] Nakayama H (2013). Delayed radiotherapy for patients with localized prostate cancer: Validation by propensity score matching. Anticancer Res..

[33] NCCN Clinical Practice Guidelines for Prostate Cancer, v. 4.2018. https://www.nccn.org/professionals/physician_gls/default.aspx#prostate. (2018). 10.1007/978-1-4614-6828-8.

[34] Grossfeld GD, Latini DM, Lubeck DP, Mehta SS, Carroll PR (2003). Predicting recurrence after radical prostatectomy for patients with high risk prostate cancer. J. Urol..

[35] D’Amico AV (1998). Biochemical outcome after radical prostatectomy, external beam radiation therapy, or interstitial radiation therapy for clinically localized prostate cancer. JAMA.

[36] Jung J-W, Lee JK, Hong SK, Byun S-S, Lee SE (2015). Stratification of patients with intermediate-risk prostate cancer. BJU Int..

[37] Keane FK (2014). The likelihood of death from prostate cancer in men with favorable or unfavorable intermediate-risk disease. Cancer.

[38] Zumsteg ZS (2013). A new risk classification system for therapeutic decision making with intermediate-risk prostate cancer patients undergoing dose-escalated external-beam radiation therapy. Eur. Urol..

[39] Bese NS, Hendry J, Jeremic B (2007). Effects of prolongation of overall treatment time due to unplanned interruptions during radiotherapy of different tumor sites and practical methods for compensation. Int. J. Radiat. Oncol..

[40] Radiologists., R. C. of. *The Timely Delivery of Radical Radiotherapy: Guidelines for the Management of Unscheduled Treatment Interruptions Fourth Edition*. (2019).

[41] de la Vega JM (2016). Effects of interruptions for public holidays in Spain on radiotherapy treatments. Phys. Med..

[42] de la Vega JM (2016). Management of interruptions to fractionated radiotherapy treatments: Four and a half years of experience. Phys. Med..

[43] Sandler HM (2018). Role of overall treatment time in the management of prostate cancer patients: How to manage unscheduled treatment interruptions. Int. J. Radiat. Oncol..

[44] Fundowicz M (2020). Multicentre clinical radiotherapy audit in rectal cancer: Results of the IROCA project. Radiat. Oncol..

[45] Danielson B (2011). Development of indicators of the quality of radiotherapy for localized prostate cancer. Radiother. Oncol..

[46] Izewska J (2018). Improving the quality of radiation oncology: 10 years’ experience of QUATRO audits in the IAEA Europe Region. Radiother. Oncol..

[47] Vaandering A, Jornet N, Scalliet P, Coffey M, Lievens Y (2018). Doing the right thing: Quality in radiotherapy, a European perspective. Radiother. Oncol..

