# Supplementary material for: Results of the IROCA international clinical audit in prostate cancer radiotherapy at six comprehensive cancer centres
Source: Sci Rep. 2021 Jun 10;11:12323. doi: 10.1038/s41598-021-91723-0 (PMC8192927; doi:10.1038/s41598-021-91723-0)

Title: **Results of the IROCA international clinical audit in prostate cancer radiotherapy at six comprehensive cancer centres**

Authors: Carla Lopes de Castro, MD^1^; Magdalena Fundowicz, MD, PhD^7^; Alvar Roselló, MD^5^; Josep Jové, MD^4^; Letizia Deantonio, MD^3^; Artur Aguiar, MD^1^_;_ Carla Pisani, MD^3^; Salvador Villà, MD^4^; Anna Boladeras, MD^6^; Ewelina Konstanty, PhD^7^; Marta Kruszyna-Mochalska, MSc^2,7^; Piotr Milecki, MD, PhD^2,7^; Diego Jurado-Bruggeman, MSc^5^; Joana Lencart, BSc^1^; Ignasi Modolell, PhD^6^; Carles Muñoz-Montplet, PhD^6^; Luisa Aliste, MPH^6^; Maria Gloria Torras, MD^6^; Montserrat Puigdemont, BSc^5^; Luísa Carvalho, MD^1^; Marco Krengli, MD^3^; Ferran Guedea, MD, PhD^6^; Julian Malicki, PhD ^2,7^

**Supplementary Table S1**

| **INDICATORS FOR PROSTATE RADIOTHERAPY** |
| --- |
|  |
| **DIAGNOSTIC AND PRE-TREATMENT PHASE** |
| % patient diagnosed at a different hospital |
| % of patients with staging MRI |
| % of patients with risk stratification (including PSA, Gleason, TNM) |
| % of patients presented to the Tumor Board |
| % of patients included in a clinical trial |
| % of patients evaluated before treatment in the RO Department clinical session |
| Time between first visit at RO department and beginning of radiotherapy (EBRT or brachytherapy) |
| Time between CT simulation and beginning of radiotherapy |
|  |
| **TREATMENT PHASE** |
| Treatment by risk group |
| % of patients with interruptions during the EBRT treatment |
| % treatment interruptions because of patients’ reasons |
| % treatment interruptions because of center related reasons (day off, technical) |
| Has the center done something to compensate this interruption? |
| % of patients who completed the treatment in the prescribed time |
| % of patients of high risk who have received long-term hormonotherapy |
| % of patients of high risk who have received brachytherapy (as a boost) |
| % of patients treated using new technologies (IMRT) |
| % of patients treated using new technologies (VMAT) |
| % of patients treated using new technologies (SBRT) |
|  |
| **FOLLOW-UP PHASE** |
| **ADVERSE EFFECTS (> grade 2)** |
| *Acute (< 6 months)* |
| % of patients with rectal bleeding |
| % of patients with rectal mucositis |
| % of patients with enterocolitis |
| % of patients with cystitis-urethritis |
| % of patients with hematuria |
| % of patients with urinary retention |
| % of patient with erectile disfunction |
| *Chronic (> 6 months)* |
| % of patients with rectal bleeding |
| % of patients with rectal mucosites |
| % of patients with enterocolitis |
| % of patients with cystitis-urethritis |
| % of patients with hematuria |
| % of patients with urinary retention |
| % of patient with erectile disfunction |
| **FOLLOW-UP** |
| Biochemical free survival |
| % Patients with regular follow-up (≥ 2 visits/year) post-treatment |
| Relapse rate |
| Mortality |

**Supplementary Table S2. Treatment phase indicators**

| **INDICATOR** | **ICO-B** | **ICO-G** | **ICO H** | **IPO** | **NO** | **WCO** | **ALL** | **P** |
| --- | --- | --- | --- | --- | --- | --- | --- | --- |
| Patients included in clinical trial, n (%) | 3 (7.5%) | 1 (2.5%) | 0 (0%) | 0 | 0 | 6 (15%) | 10 (4.2%) | 0.003 |
| Patient presented at RO department clinical session prior to treatment, n (%) | 40 (100%) | 40(100%) | 30 (75%) | 0 | 40 (100%) | 0 | 150(62.5%) | <0.001 |
| Median time (days) between initial visit to RO department and initiation of radiotherapy | 102.5 | 83.5 | 66 | 42 | 77 | 70 | 78 | <0.05 |
| Median time (days) between  CT simulation and initiation of radiotherapy. | 15.5 | 17.5 | 33 | 14 | 19.5 | 12 | 18 | <0.001 |

Abbreviations: EBRT, external beam radiotherapy; BT, brachytherapy; RO, radiation oncology; WCO, Wielkopolskie Centrum Onkologii; IPO, Instituto Português de Oncologia (IPO) in Porto, Portugal; NO, Università degli Studi del Piemonte Orientale; ICO-H, Institut Català d'Oncologia, Hospitalet, Spain; ICO-B (Badalona), and ICO-G (ICO-Girona).

**Supplementary Table S3. Patients treated with brachytherapy (combined or alone) and dose received.**

| **CENTRE** | **Patients, n (%)** | **Dose** | | | | |
| --- | --- | --- | --- | --- | --- | --- |
|  |  | 9 Gy | 10 Gy | 15 Gy | 31.5 Gy | 145 Gy |
| **ICO-H** | 20 (50%) | 10 | 0 | 0 | 0 | 10 |
| **IPO** | 12 (60%) | 0 | 1 | 0 | 0 | 11 |
| **WCO** | 21 (52.5%) | 0 | 0 | 11 | 6 | 4 |
| **All** | 53 (22.1%) | 10 | 1 | 11 | 6 | 25 |

Abbreviations: Wielkopolskie Centrum Onkologii; IPO, Instituto Português de Oncologia (IPO) in Porto, Portugal; ICO-H, Institut Català d'Oncologia, Hospitalet, Spain.

**Supplementary Table S4.** **Treatment-related indicators**

| **INDICATOR** | | **ICO-B** | **ICO-G** | **ICO H** | **IPO** | **NO** | **WCO** | **ALL** | |  |
| --- | --- | --- | --- | --- | --- | --- | --- | --- | --- | --- |
|  |  |  |  |  |  |  |  | **n** | **%** | P |
| **Treatment interruptions ≥ 1 day during EBRT, n (%)** | | 39  (97.5%) | 39  (97.5%) | 29  (72.5%) | 16  (40%) | 35  (87.5%) | 3  (7.5%) | **161** | **67.1** | <0.001 |
| **Compensation for treatment interruption, yes (%)** | | 0 | 0 | 0 | 14  (87.5%) | 0 | 2  (66.7%) | **16** | **9.9** | 0.011 |
| **Patients completing EBRT in the prescribed time (%)** | | 7  (17.5%) | 2  (5%) | 3  (10%) | 21  (72.4%) | 6  (15%) | 18  (60%) | **57** | **27.3** | n/a |
| **Patients completing EBRT treatment in the prescribed time (± 4 days) (%)** | | 22  (55%) | 18  (45%) | 13  (43.3%) | 29  (100%) | 34  (85%) | 24  (80%) | **140** | **67** | <0.05 |
| **Boost BT in high-risk patients, yes (%)** | | 0 | 0 | 9  (60%) | 1  (5.9%) | 0 | 11  (50%) | **21** | **17.5** | <0.001 |
| **IMRT or VMAT (%)** | | 17  (42.5%) | 24  (60%) | 28  (93.3) | 29  (100%) | 40  (100%) | 17  (57.6%) | **155** | **74.2** | n/a |
| **SBRT (%)** | | 0 | 0 | 1  (3.3%) | 0 | 0 | 11  (27.5%) | **12** | **5.7** | n/a |
| **3D-RT (%)** | | 23  (57.5%) | 15  (37.5%) | 0 | 0 | 0 | 0 | **38** | **18.2** | n/a |
| **Image guidance technique** | **kV/MV** | 24 | 2 | 0 | 0 | 19 | 1 | **46** | **22** | n/a |
|  | **CBCT** | 0 | 0 | 3 | 4 | 21 | 6 | **34** | **16.3** | n/a |
|  | **CBCT + kV** | 16 | 0 | 13 | 3 | 0 | 11 | **43** | **20.6** | n/a |
|  | **Fiducial+KV or MV** | 0 | 33 | 11 | 0 | 0 | 11 | **55** | **26.4** | n/a |
|  | **Fiducial + CBCT** | 0 | 0 | 2 | 0 | 0 | 0 | **2** | **1** | n/a |
|  | **Fiducial+CBCT+kV** | 0 | 5 | 1 | 22 | 0 | 0 | **28** | **13.4** | n/a |
|  | **Missing** | 0 | 0 | 0 | 0 | 0 | 1 | **1** | **0.5** | n/a |

Abbreviations: EBRT, indicates external beam radiotherapy; IMRT, intensity-modulated radiotherapy; VMAT, volumetric arc radiotherapy; SBRT, stereotactic body radiotherapy; IGRT, image-guided radiotherapy; BT, brachytherapy; HT, hormonotherapy; CBCT, cone-beam computed tomography; kV/MV kilovoltage-megavoltage. WCO, Wielkopolskie Centrum Onkologii; IPO, Instituto Português de Oncologia (IPO) in Porto, Portugal; NO, Università degli Studi del Piemonte Orientale; ICO-H, Institut Català d'Oncologia, Hospitalet, Spain; ICO-B (Badalona), and ICO-G (ICO-Girona).

**Supplementary Table S5. Treatment-related adverse effects**

|  | | | **ICO-B** | | **ICO-G** | | **ICO H** | | | **IPO** | | | **NO** | | **WCO** | | **ALL** | | **P value** |
| --- | --- | --- | --- | --- | --- | --- | --- | --- | --- | --- | --- | --- | --- | --- | --- | --- | --- | --- | --- |
|  | | | *n* | % | *n* | % | *n* | % | | *n* | | % | *n* | % | *n* | % | ***n*** | **%** |  |
| **Treatment-related AEs registered on the medical record** | Yes  No AE/missing | | 37  3 | 92.5  7.5 | 33  7 | 82.5  17.5 | 34  6 | 85  15 | | 18  22 | | 45  55 | 32  8 | 80  20 | 3  37 | 7.5  92.5 | **157**  **83** | **65.4**  **34.6** | <0.001 |
| **Acute adverse effects (<6 months) > grade 2** | | | | | | | | | | | | | | | | | | |  |
|  | | | *n* | | *n* | | *n* | | | | *n* | | *n* | | *n* | | ***n*** | ***%*** |  |
| **Rectal bleeding** | | | 1 | | 0 | | 0 | | | | 0 | | 1 | | 1 | | **3** | **1.3** | *0.694* |
| **Rectal mucositis** | | | 0 | | 0 | | 0 | | | | 0 | | 0 | | 3 | | **3** | **1.3** | *0.028* |
| **Enterocolitis** | | | 0 | | 0 | | 0 | | | | 0 | | 0 | | 2 | | **2** | **0.8** | *0.073* |
| **Cystitis-urethritis** | | **Yes**  **Missing** | 29 | | 24 | | 11 | | | | 2 | | 9  23 | | 7 | | **82** | **34.2** | <0.001 |
| **Hematuria** | | | 0 | | 0 | | 2 | | | | 0 | | 0 | | 0 | | **2** | **0.8** | *0.073* |
| **Urinary retention** | | | 1 | | 0 | | 0 | | | | 3 | | 1 | | 0 | | **5** | **2.1** | *0.137* |
| **Erectile dysfunction** | | **Yes**  **Missing** | 10  18 | | 16  14 | | 0  30 | | | | 1  0 | | 0  39 | | 0  1 | | **27** | **11.3** | <0.001 |
| **Chronic adverse effects (<6 months) > grade 2*** | | | | | | | | | | | | | | | | | | |  |
| **Rectal mucositis** | | | 0 | | 0 | | 0 | | 0 | | | | 2 | | 0 | | **2** | **0.8** | n/a |
| **Cystitis-urethritis** | | | 5 | | 5 | | 2 | | 3 | | | | 1 | | 6 | | **22** | **9.2** | n/a |
| **Hematuria** | | | 0 | | 0 | | 0 | | 1 | | | | 1 | | 0 | | **2** | **0.8** | n/a |
| **Urinary retention** | | | 0 | | 0 | | 0 | | 1 | | | | 0 | | 1 | | **2** | **0.8** | n/a |
| **Erectile dysfunction** | | | 11 | | 0 | | 1 | | 2 | | | | 0 | | 1 | | **15** | **6.3** | n/a |

Abbreviations: AE, adverse effects. WCO, Wielkopolskie Centrum Onkologii; IPO, Instituto Português de Oncologia (IPO) in Porto, Portugal; NO, Università degli Studi del Piemonte Orientale; ICO-H, Institut Català d'Oncologia, Hospitalet, Spain; ICO-B (Badalona), and ICO-G (ICO-Girona).

*There were no reported cases of chronic rectal bleeding or enterocolitis.

**SUPPLEMENTARY FIGURES**

Supplementary Figure S1. Distribution of risk groups by institution. The numbers in each column represent the number of patients at each institution according to risk category (high, intermediate, low)

Supplementary Figure S2. Number of patients at each institution who received EBRT or BT

Supplementary Figure S3. EBRT dose administered and number of patients receiving that dose at each institution

Supplementary Figure S4. Dose per fraction (Gy) by institution in patients who received EBRT

**FIG S1**

**
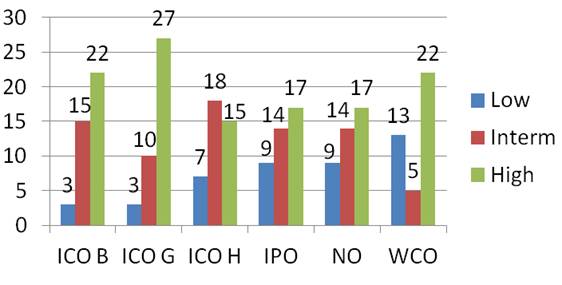
**

**FIG S2**

**
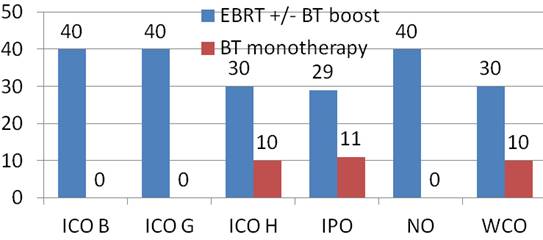
**

**FIG S3**

**
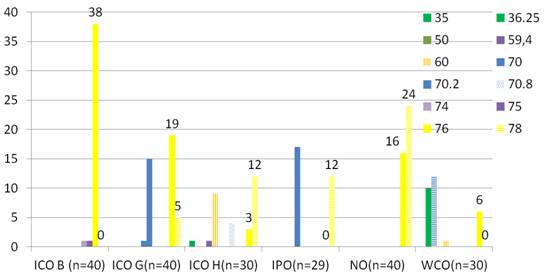
**

**FIG S4**


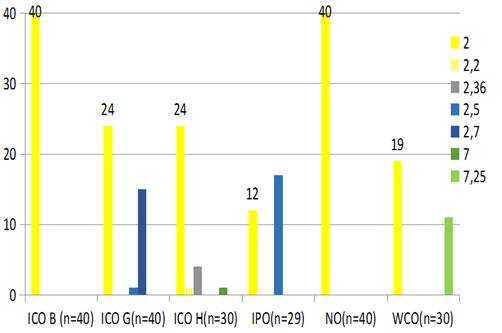

Supplement: Supplementary file 1 — Supplementary Information 1. [file 41598_2021_91723_MOESM1_ESM.docx]
